# 

**Published:** 1974-10

**Authors:** 


Bristol Medico-Chirurgical Journal Vol. 89 (iv) No. 332

### Contents

'Through a Glass Darkly' (The Development of Needle Aspiration Biopsy) ... ... 59
A. J. Webb

Learning to Care for the Mentally Handicapped ... ... ... ... ... ... 69
Valerie A. Cowie

Solitary Cyst of the Pancreas and 'Reversible Diabetes Mellitus' ... ... ... 71
A. A. J. Barros d'Sa

South West Orthopaedic Club ... ... ... ... ... ... ... ... 75

The Medical Library ... ... ... ... ... ... ... ... ... 76

University of Bristol ... ... ... ... ... ... ... ... ... 77

Printed by F. Bailey & Son Ltd., Dursley, Glos.

